# Three Temperature Regimes in Superconducting Photon Detectors: Quantum, Thermal and Multiple Phase-Slips as Generators of Dark Counts

**DOI:** 10.1038/srep10174

**Published:** 2015-05-19

**Authors:** Andrew Murphy, Alexander Semenov, Alexander Korneev, Yulia Korneeva, Gregory Gol’tsman, Alexey Bezryadin

**Affiliations:** 1 Department of Physics, University of Illinois at Urbana-Champaign, Urbana, Illinois 61801, USA; 2 Moscow State Pedagogical University, 1 Malaya Pirogovskaya, 119991 Moscow, Russia; 3 Moscow Institute of Physics and Technology, 141700, Dolgoprudny, Moscow Region, Russia; 4 National Research University Higher School of Economics, Moscow 101000, Russia

## Abstract

We perform measurements of the switching current distributions of three *w ≈ *120 nm wide, 4 nm thick NbN superconducting strips which are used for single-photon detectors. These strips are much wider than the diameter of the vortex cores, so they are classified as quasi-two-dimensional (quasi-2D). We discover evidence of macroscopic quantum tunneling by observing the saturation of the standard deviation of the switching distributions at temperatures around 2 K. We analyze our results using the Kurkijärvi-Garg model and find that the escape temperature also saturates at low temperatures, confirming that at sufficiently low temperatures, macroscopic quantum tunneling is possible in quasi-2D strips and can contribute to dark counts observed in single photon detectors. At the highest temperatures the system enters a multiple phase-slip regime. In this range single phase-slips are unable to produce dark counts and the fluctuations in the switching current are reduced.

Quantum tunneling between macroscopically distinct states has been studied extensively in Josephson junctions and nanowires^1^,^2^. Some of the most basic evidence for macroscopic quantum tunneling (MQT) in these systems comes from the saturation of the standard deviation of switching current distributions and the saturation of escape temperature at bath temperatures below some threshold value^1^,^3^,^4^,^5^,^6^. Exploring quantum tunneling in quasi-2D superconductors has recently become a particularly interesting topic as the practical use of quasi-2D NbN strips as single photon detectors has grown^7^,^8^,^9^. In practice, these detectors are driven at currents near their critical currents so that when a photon strikes the superconductor, it can cause a segment of the strip to become normal for a short period of time, registering a voltage pulse. However there also exists a rate of false events, known as dark counts, whereby a voltage pulse can be detected without an incident photon. A possible origin of these dark counts is thermally activated escape from the superconducting state^10^,^11^,^12^,^13^,^14^,^15^. Such thermal dark counts can be suppressed by reducing temperature. A second possible origin of dark counts is MQT^16^ of vortices or phase-slips between the ends of the quasi-2D strip^10^,^11^,^12^,^13^,^14^,^15^,^16^,^17^. This option has been speculated theoretically but not yet observed experimentally. The rate of MQT is not expected to be strongly affected by temperature, and therefore MQT could provide a base-level dark count rate which can be achieved if the photon detector is cooled below some threshold quantum temperature. Note that we understand a phase-slip event to be any process which leads to a quantized phase change of the order parameter by 2*π* between the ends of the strip, which represents the elementary dissipative event in the superconductor.

As dark counts in wide strips have been investigated, a debate over the microscopic process by which the strips switch from the superconducting state to the normal state has emerged. Recent theories and experiments have supported three different escape processes (all of which are 2*π* phase-slip events), including single vortices crossing an edge barrier^10^,^11^,^12^,^18^, vortex-antivortex pairs splitting apart under the action of the Lorentz force^11^,^13^,^14^,^15^ and escape through an energy saddle point which does not involve a vortex core^19^,^20^,^21^. Such a saddle point, with a suppressed but still above zero order parameter, has been used to explain Little-type phase-slips in one-dimensional superconducting wires^22^,^23^. Recently, it was generalized theoretically to quasi-2D superconducting strips^20^,^21^. After passing through the energy saddle point, i.e. when the free energy has passed through the maximum and is already lowered, a vortex is formed in the strip. The Lorentz force then pushes the vortex across the strip causing a phase-slip. So in any theory, it is expected that a vortex or vortex-antivortex pair is needed to produce a phase-slip. Yet in the vortex-free saddle point model, the maximum of the free energy is achieved before the vortex core is created. For the case of MQT, this model is most plausible because it does not involve quasiparticle dissipation. Note that according to Caldeira-Leggett theory any dissipation reduces the rate of quantum tunneling exponentially^24^. Another possible cause of dark counts could be multi-phase-slip switching events, or phase diffusion, which have been observed in many superconducting devices including one-dimensional wires^2^,^3^,^25^,^26^,^27^ but have not been investigated so far as a candidate for the dark counts in photon detectors.

Most experiments so far were performed in the range of intermediate temperatures, where superconducting single-photon detectors commonly operate and MQT is not expected to be observed. The few experiments which have reached much lower temperatures have drawn conflicting conclusions on both escape method and on presence of MQT^10^,^13^,^17^. It has therefore become apparent that in order to study MQT in quasi-2D strips, evidence for MQT should come from new sources such as counting-statistics as historically was done for Josephson junctions and one-dimensional wires.

In this article, we report evidence of macroscopic quantum tunneling in wide (compared to the size of the vortex core) NbN strips by demonstrating the saturation of the standard deviation of their switching current distributions and the saturation of the escape temperature. All measurements are done on commercially available superconducting photon detectors, made with quasi-2D superconducting meandering strips. Because the switching events are observed without any photon irradiation and with careful multi-stage electromagnetic noise filtering, these events represent so-called “dark counts”. We fit our data using the general Kurkijärvi-Garg (KG)^28^ model and show that the escape rates in such systems can be approximated using forms similar to those derived for Josephson junctions, nanowires and graphene junctions made superconducting by proximity effect^4^,^27^,^28^,^29^,^30^,^31^. We also establish that at higher temperatures single phase-slips cannot switch our superconducting strips. Switching events and therefore dark counts are due to an approximate coincidence of more than one phase-slip at high temperatures.

## Experiment

**Figure 1:**
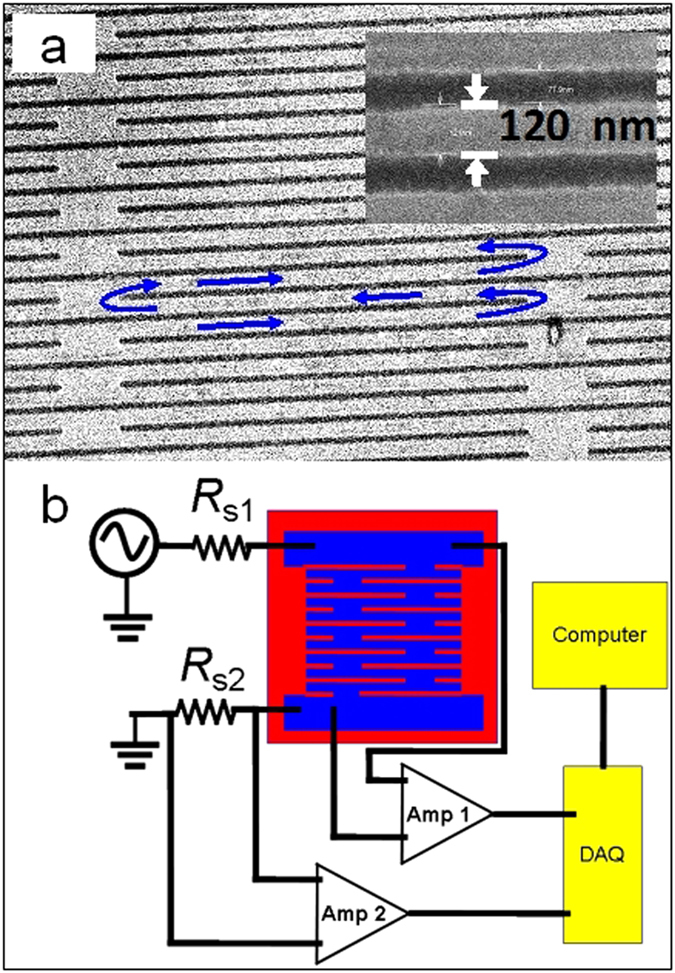
An SEM image of a niobium nitride photon detector and schematic diagram of the setup. (**a**) An SEM image of Sample 4. The NbN superconducting strip (lighter region) is roughly 250 *μ*m in length and about 4 nm thick. The image is stretched vertically to make the strip more pronounced. The distance between the current turning points is about 7 *μ*m, and the wide turns in the pattern allow the current to change direction without creating regions of high average current density. The path which the current travels is indicated by arrows. Not shown in the figure, to the far right or the far left, each horizontal NbN segment comes to an abrupt end and has no additional connections to the rest of the superconductor. The inset is a zoomed in view on one segment of the detector showing the width of the strip to be about 120 nm. (**b**) A schematic diagram of the setup. Superconducting NbN, shown in blue, is placed on a Si chip, which is shown in red. Voltage is measured directly across the sample. The voltage across 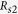 = 1 kΩ is used to determine the current though the strip using Ohm’s law. The resistor 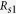 = 46 kΩ serves to limit the current in the circuit. Signals are amplified, read by a data acquisition (DAQ) card, and analyzed by computer.

The photon detectors measured are approximately 245 *μ*m long, 4 nm thick and 120 nm wide NbN strips (Fig. 1a). The coherence length *ξ* and perpendicular magnetic penetration depth 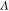 of similar samples are known to be around 5 nm and 50 *μ*m respectively^32^,^33^. Because 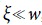, the strip forming the photon detector is considered quasi-two-dimensional. Yet the supercurrent is expected to be uniform across the width of the strip because 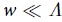. Note that the average current density is lower at the turning regions because, according to the employed sample design (Fig. 1a), the width of the strip is roughly twice larger near the turning region. Thus it is not expected that phase-slips would occur predominately at the turning regions, rather than at other possible imperfections or constrictions along the current path.

**Figure 2:**
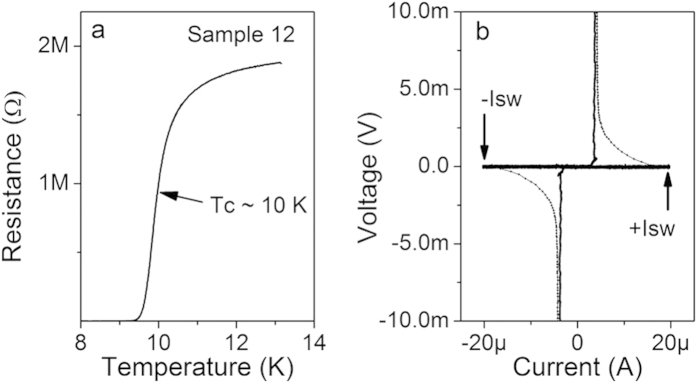
Critical temperature and switching current. (**a**) The RT curve of Sample 12 shows a critical temperature around 10 K. (**b**) A typical VI curve. The sample resistance is much larger than the series resistance, thus the current through the sample quickly decreases when the sample becomes resistive.

The samples are fabricated from 4 nm thick niobium nitride (NbN) film. The critical temperature of each device is around 10 K (Fig. 2a). The fabrication procedure is similar to the one described in detail in Ref. 34. In brief, the NbN film is deposited by DC reactive magnetron sputtering on thermally oxidized silicon wafers. The film is patterned as meander-shaped strip by electron beam lithography in PMMA 950 K resist and reactive ion etching in SF_6_. The strips outside the meander are used for proximity effect correction in e-beam lithography.

Measurements were performed in a He-4 system with a base temperature of 1.45 K. A diagram of the setup is shown in Fig. 1b. The function generator was connected to the photon detector across a series resistor of value 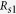 = 46 kΩ; the other end of the detector was connected to the ground through a series resistor of value 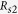 = 1 kΩ. The normal state resistance of our devices *R*_*n*_ was on the order of 1 MΩ. The voltage was measured across the detector directly while the current was determined by measuring the voltage across the 1 kΩ series resistor. A sinusoidal bias voltage exceeding the necessary voltage to reach the critical current of the photon detector was applied to the system. Signal lines were filtered by pi-filters at high temperature and passed through silver-particle filters at the base temperature. In the experiments, the bias current is slowly increased from zero and the voltage and the current are carefully monitored. As the current reaches some critical value called the switching current *I*_*sw*_, the voltage across the photon detector suddenly jumps from zero to a rather large value and the current through the system suddenly decreases. This happens because the photon detector switches to the resistive state, and the normal state resistance of the detector is much larger than the series resistors. The value of current at the moment of the voltage jump, which corresponds to the peak current measured, was recorded as *I*_*sw*_. Such measurements were repeated at least 10,000 times at each temperature for Samples 4 and 12, and 50,000 times for Sample 7. The peak bias voltage was adjusted at each temperature in order to keep the sweep speed ν_I_ = dI/dt at the switching current constant with temperature. Note that the current was changing with time as 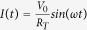 where *V*_0_ is the peak bias voltage, *R*_*T*_ = 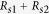 is the total resistance of the experimental setup while the sample is superconducting, and *ω* = 41 Hz is the frequency. Therefore the sweep speed at *I = I*_*sw*_ can be calculated as 
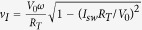
. Sweep speeds at *I*_*sw*_ were kept near 3 mA/s, 3.6 mA/s and 2.6 mA/s for Samples 4, 7 and 12 respectively.

## Analysis

Figure 2b shows a typical voltage versus current dependence^35^ (VI curve). When a segment of the NbN strip switches from superconducting to normal, the total resistance of the system sharply increases, causing the current through the system to fall. The switching current is marked in Fig. 2b with arrows. In fact, there are two such switching currents, one occurring at the positive bias (*I*_*sw*_*+*) and one occurring at the negative bias (*I*_*sw*_−).

**Figure 3:**
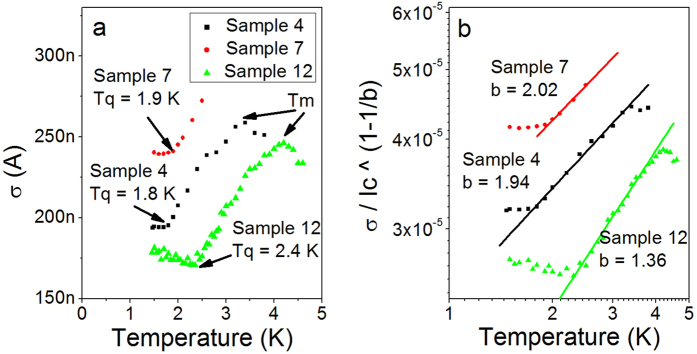
Standard deviations of switching distributions. (**a**) The standard deviations of the switching current distributions of Samples 4, 7 and 12 are plotted vs temperature. All three samples show saturation at low temperatures which is known as a signature of macroscopic quantum tunneling. The temperatures at which *σ* saturates are recorded as the quantum temperatures of the strips and are indicated on the plot as *T*_*q*_. The multiple phase-slip regime is visible at *T > T*_*m*_. It is manifested by a drop of the standard deviation with increasing temperature. (**b**) Fits of 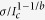 to *T*^*b*^ (solid curves) are performed for *T*_*q*_* < T < T*_*m*_ in order to find the best-fit power, *b*, for the temperature dependence according to the Kurkijärvi analysis^29^. The current dependence in the energy barrier for a phase-slip event has the same power (see Eq. 2). The best-fit powers are shown in the figure and also listed in Table 1. The data and fit for Sample 12 in Figure b are multiplied by a factor of 9 so that all three curves can be easily seen.

At any given temperature, the switching current exhibits a stochastic nature, resulting in a distribution of switching currents. The standard deviation *σ* of these distributions, which is plotted versus temperature in Fig. 3a, can be analyzed to determine whether quantum or thermal fluctuations are responsible for the strip switching to the resistive state^5^,^28^,^29^. At high bath temperatures (*T > T*_*m*_ where T_*m*_ is the temperature corresponding to the maximum in *σ*) standard deviation decreases with increasing temperature. At these temperatures, multiple thermally activated phase-slips (TAPS) are necessary to switch the strip to a resistive state. The multiple phase-slip regime is such that a single phase-slip cannot switch the quasi-2D strip to a resistive state, and an almost perfect coincidence in time and in space of two or more phase-slippage events is necessary to produce a switching event. According to previous studies the main signature of the multiple phase-slip regime is the fact that *σ* decreases with increasing temperature^2^. In the intermediate temperature range, at *T*_*q*_* < T < T*_*m*_, single TAPS are responsible for switching events, and the standard deviation decreases with cooling^1^,^29^. At the lowest temperatures the standard deviation saturates, indicating that quantum tunneling of phase-slips (QPS) is the dominant process by which the system escapes from the metastable superconducting state. The temperatures at which the standard deviation saturates are taken as the quantum crossover temperatures *T*_*q*_ and recorded in . Thus we find evidence of MQT in quasi-2D systems and determine the transition temperature *T*_*q*_ between TAPS and QPS in photon detectors. Reducing the bath temperature down to *T*_*q*_ is expected to reduce the dark count rate to its quantum minimum and thus can be used to make the detectors more efficient.[Table t1]

The observed crossover at *T = T*_*m*_ (see the discussion above) suggests that in the normal temperature range in which photon-detectors are operated, T ≈ 5 K, multiple TAPS are responsible for dark counts. Therefore existing theoretical models describing dark counts would be made more accurate if they included the multiple TAPS scenario^2^,^25^,^26^. When *σ* is small, small changes in applied current return large changes in the average dark count rate. Therefore it should be advantageous to operate photon detectors far from T_*m*_, i.e., as far as possible from the temperature where the fluctuations in the switching current are most noticeable. It should also be advantageous to reduce T_*m*_, thereby reducing σ in the multiple TAPS regime.

**Figure 4:**
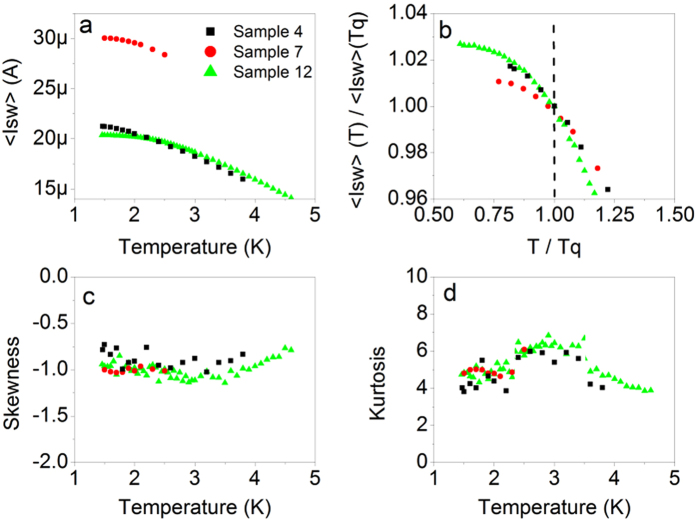
Switching distribution statistics. The (**a**) mean switching currents and (**b**) normalized mean switching currents of Samples 4, 7 and 12 increase with cooling in all temperature regions, including *T < Tq*. The normalized mean switching current is obtained by dividing the mean switching current by its value at 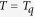 and the normalized temperature is obtained by dividing *T* by *T*_*q*_. The (**c**) skewness and (**d**) kurtosis reflect expected values and verify that the saturation in standard deviation is not caused by reaching a base level of noise in the system. At 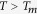 skewness and kurtosis trend toward Gaussian distribution values. The legend presented in (**a**) pertains to all four plots in this figure.

While the standard deviation saturates at low temperatures, the mean switching current, *<I*_*sw*_*>*, shown in Fig. 4a, does not. To make this trend clearer we normalize *<I*_*sw*_*>* by its value at *T*_*q*_, normalize the temperature by *T*_*q*_ and focus on the lowest temperatures in Fig. 4b. Analyzing the mean switching current provides a check that the saturation seen in the standard deviation is not simply due to a saturation of sample temperature, because we would expect to see a similar saturation in the mean switching current in this case. In fact, what is observed is that *σ* shows a sharp saturation at *T*_*q*_ while *<I*_*sw*_*>* does not exhibit any peculiarity at *T*_*q*_ at all. This type of behavior is expected from the Kurkijärvi-Garg model^4^, and it confirms our initial conclusion that the observed saturation at *T = T*_*q*_ is the crossover to MQT.

**Figure 5:**
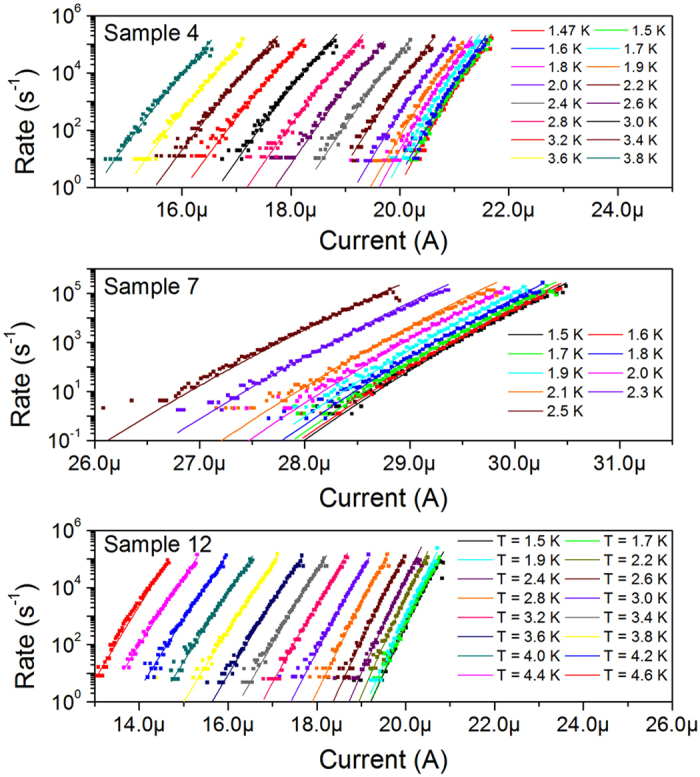
Rates of dark counts. The rates of dark counts (symbols) for Samples 4, 7 and 12 are plotted vs current and fitted (solid lines) using the KG model for thermal activation. Fit parameters are shown in Table 1 and Fig. 6. The lowest temperature curves correspond to the highest currents. At low temperatures, the average value of the switching current changes slowly and the rate curves become more difficult to distinguish in these plots.

At *T < T*_*m*_, the skewness of the switching distributions is about −1 and kurtosis is about 5, as shown in Fig. 4c,d. It has been shown that these values are expected for switching distributions in the temperature range where escape is caused by single phase-slips, whether quantum or thermal^36^. Typical perturbations, such as noise in the setup or an influx of external photons would pull skewness toward zero and kurtosis toward three, corresponding to a Gaussian distribution^36^. Skewness and kurtosis can also be used to detect the temperatures at which multiple phase-slips must be responsible for escape, as distributions become increasingly Gaussian for *T > T*_*m*_. This trend can be seen in Sample 4 and Sample 12. Impacts from high-energy cosmic rays or products of radioactive decay events that can cause dark counts in superconducting kinetic-inductance detectors^37^,^38^ can additionally be ruled out as the source of dark counts in our setup because they result in a much slower dependence of switching rates on bias current than those found in Fig. 5. The upper limit for the rate of the events with high energy transferred to the detector (greater than of visible-light photon) was 10^−4^ s^−1^ 39. Therefore we conclude that our switching measurements are dominated by thermal or quantum phase-slips.

Switching rates were calculated by performing the Kurkijärvi-Fulton-Dunkleberger (KFD) transformation^29^,^40^ on the switching current distributions, and fit using the Kurkijärvi-Garg model^4^,^28^,^29^,^30^. The switching currents are binned with bin size *ΔI* = 30 nA. The first bin is chosen such that it contains the lowest switching current measured for the particular sample and temperature. Subsequent bins are placed adjacent to one another at increasing currents until the highest switching current for the particular sample and temperature has been exceeded, resulting in a total of *N* bins. The total number of switching events 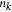 contained within bin *k* are evaluated at the bin center 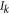. The KFD transformation takes the binned distribution of switching events and returns the rate of switching events evaluated at the bin centers. The rate Γ at some bin center *I*_*j*_ is given by


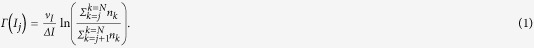


We assume that the energy barrier for thermal escape can be approximated in the general form of


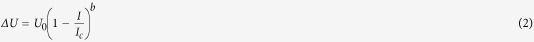


where 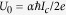 is the energy barrier at zero current. Here, 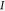 is the applied current, 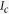 is the temperature-dependent critical current and *e* is the charge of an electron. The positive constants *a* and *b* are used as fitting parameters. This form allows our results to be easily compared to Josephson junctions and long wires. In the LAMH model of long wires^3^,^23^,^41^,^42^, *a* = 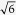 and *b* = 5/4. For Josephson junctions, *a* = 4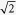/3 and *b* = 3/2^28^. At high currents the energy barrier for a phase-slip is expected to become narrow. We can roughly estimate the width of the energy barrier from the energy of a vortex core, 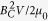, and the work done by the Lorentz force on a vortex, 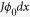, where *d* is the film thickness, *J* is the supercurrent density, 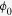 is the flux quantum, and *x* is the distance of the vortex core measured from the edge of the strip. 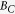 is the critical magnetic field, and V is the volume of the vortex, which we will estimate as 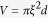 where *ξ* is the coherence length^3^. The energy barrier reaches zero when the core energy equals the work performed by the Lorentz force at 
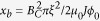
. Using equation 4.20 from Ref. 3, our estimate can be rewritten as 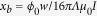. Plugging in a typical value of current, *I = 20 μ*A, we find 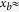  4 nm. Physically, 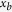 represents a rough estimate for the barrier width for a phase-slip tunneling event in our quasi-2D superconducting strip. After crossing this energy barrier, the system will develop a vortex at 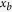 which will then move classically, under the action of the Lorentz force, across the strip causing a phase-slip. Because the relevant region for phase-slippage is on the order of the coherence length, our system should be able to be described similarly to a quasi-one-dimensional wire, thus justifying our choice of the barrier form (Eq. 2). We note that detailed theory^20^ also supports the idea that at high currents the energy saddle point is located near the edge of the film, effectively reducing the problem to a quasi-one-dimensional case.

Using the Arrhenius activation equation, we fit the rates to


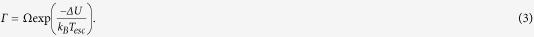


Ω is the attempt frequency, 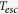 is the escape temperature, which may or may not equal the sample temperature, and 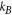 is the Boltzmann constant.

The critical current was determined at each temperature by following the KG model^28^,^29^,^30^ 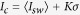 where 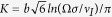 and 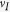 is the sweep speed. The only temperature-dependent variables in the equation for 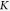 are found in the logarithm, and therefore *K* is expected to vary slowly. The actual value of *K* varies slowly with temperature within 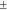
1 of the values listed in Table 1.

For simplicity, we ignore any current or temperature dependence in the attempt frequency. It should be noted that the attempt frequency often appears within a logarithm in our analysis and therefore small changes in Ω should not have a large effect on our results. Additionally, we have observed that good fits can be obtained with different values of the attempt frequency. We therefore choose to show fits at Ω = 1 THz, on the order of the natural frequency of the superconducting gap oscillations in NbN^43^.

To find the power *b*, we make use of the second relationship in the KG model^4^,^28^,^29^,^30^


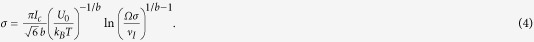


If we assume that the logarithmic term varies slowly, we can derive the relationship 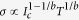, because 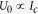. We use this relationship to determine a best-fit value of 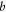 for the data corresponding to thermal activation by single phase-slips, i.e. 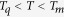. Fits are shown in Fig. 3b and best-fit values of 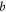 are listed in Table 1. The best-fit values of *b* found are of the same order of magnitude as predicted for thin wires and/or Josephson junctions. The reason for this similarity is that the energy saddle point is located near the edge of the strip at high bias currents, thus making the strip in many ways similar to a thin superconducting wire^20^.

**Figure 6:**
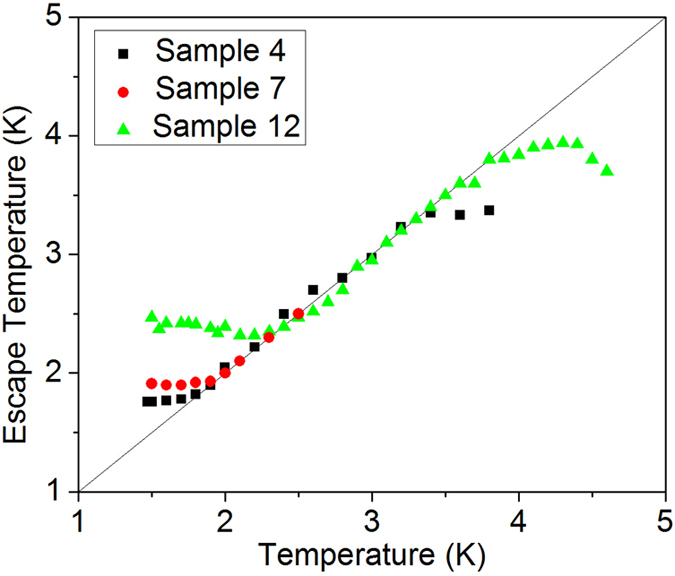
Escape temperature. Escape temperature is plotted vs real temperature for Samples 4, 7 and 12. The escape temperature was determined by fitting rate curves to Equation 3. Escape temperature was the only fitting parameter allowed to change with temperature for each sample measured. The line 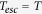 is plotted for comparison. Saturation of 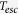 at low temperatures is a signature of MQT. In the multiple TAPS regime, 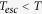.

After the critical current and the power *b* have been determined, the constant *a* is set as a temperature independent parameter and is chosen such that the rates curves calculated by Eq. 3 fit the data well for 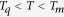. The escape temperature is then adjusted at all temperatures to produce best-fit curves. The best-fit escape temperature values are plotted versus bath temperature in Fig. 6. The rate curves, shown in Fig. 5, fit the data well using 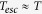 for 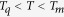, but at low temperatures best-fits require 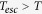. This is a signature of the crossover from thermal activation to quantum tunneling escape processes. The temperatures at which the escape temperature saturates, 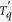 are very similar to the values of *T*_q_. Values of 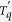 are recorded in . For all three samples, saturation of escape temperature occurs around 2 K. When multiple TAPS are responsible for dark counts, i.e. at 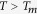, fits require 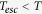 (see Ref. 27).

**Figure 7:**
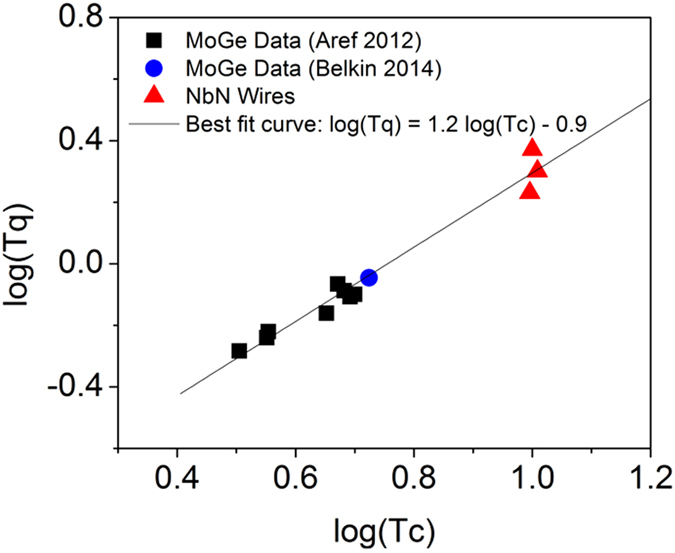
Quantum temperature versus critical temperature. The logarithm of *T*_*q*_ is plotted vs the logarithm of *T*_*c*_ along with data from MoGe samples^4^,^44^. There is a general trend that *T*_*q*_ increases with 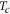. The best-fit relationship from the combined data is 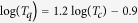.

Previous work^4^ has argued for a linear relationship between quantum and critical temperatures. In Fig. 7 we combine our data for NbN strips with data from MoGe wires and find a best-fit relationship 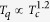. Thus we confirm the expectation that the relationship is near linear. For this fit, we used the *T*_q_ determined from the standard deviation curves of our NbN strips, although using *T*_q_′ for this fit would not give a significantly different result. It is important to emphasize that the result shown as a blue circle was obtained using a microwave setup normally used to measure superconducting qubit^44^. Its noise level is low and the whole setup is qualitatively different compared to the DC measurement setups. Thus the coincidence of the quantum temperatures confirms the conclusions that the observed crossover is not due to any uncontrolled noise but due to the internal quantum fluctuations occurring in the nanowires and thin films.

## Conclusions

We have demonstrated that dark counts in superconducting photon detectors biased at currents near the critical current are dominated by multiple phase-slips at high temperatures and macroscopic quantum tunneling at low temperatures. By observing the first four moments of the switching distributions, we have checked robustly for noise and other sources of measurement error. We find that quantum tunneling overtakes thermal activation as the dominant process for phase-slips around 2 K in all of our samples, and that the escape rate can be written in the same general form as is done for thin superconducting wires and as well as Josephson junctions, i.e. in terms of the Kurkijärvi-Garg model.

## Author Contributions

A.S., A.K., Yu.K. and G.G. fabricated the samples. A.M. and A.B. performed measurements. All authors participated in analysis, discussion and preparing the manuscript.

## Additional Information

**How to cite this article**: Murphy, A. *et al*. Three Temperature Regimes in Superconducting Photon Detectors: Quantum, Thermal and Multiple Phase-Slips as Generators of Dark Counts. *Sci. Rep.* **5**, 10174; doi: 10.1038/srep10174 (2015).

## Figures and Tables

**Table 1 t1:** Sample parameters and fitting parameters.

**Sample**	***T***_***c***_ **(K)**	***T***_***q***_ **(K)**	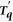	***b***	***α***	***I***_***sw***_ **at 1.5 K (** 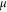 **A)**	***I***_***c***_ **at 1.5 K (** 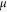 **A)**	***Rn*** **(M** 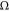 )	***K***
4	9.9	1.8	1.7	1.94	1.16	21.2	26.5	1.6	27
7	10.2	1.9	1.9	2.02	1.19	30.0	36.7	1.3	28
12	10	2.4	2.3	1.36	1.08	20.4	23.9	1.9	19
